# Roles of Integrins in Gastrointestinal Cancer Metastasis

**DOI:** 10.3389/fmolb.2021.708779

**Published:** 2021-11-15

**Authors:** Sicong Hou, Jiaxin Wang, Wenqian Li, Xin Hao, Qinglei Hang

**Affiliations:** ^1^ Department of Gastroenterology, Affiliated Hospital of Yangzhou University, Yangzhou University, Yangzhou, China; ^2^ Department of Clinical Medicine, Medical College, Yangzhou University, Yangzhou, China; ^3^ Department of Experimental Radiation Oncology, The University of Texas MD Anderson Cancer Center, Houston, TX, United States

**Keywords:** integrin, EMT, transcription factors, cell signalings, gastric cancer, colorectal cancer

## Abstract

Integrins are a large family of heterodimeric transmembrane receptors which mediate cell adhesion and transmit signals to the cell interior. The mechanistic roles of integrins have long been an enigma in cancer, given its complexity in regulating different cellular behaviors. Recently, however, increasing research is providing new insights into its function and the underlying mechanisms, which collectively include the influences of altered integrin expression on the aberrant signaling pathways and cancer progression. Many studies have also demonstrated the potentiality of integrins as therapeutic targets in cancer treatment. In this review, we have summarized these recent reports and put a particular emphasis on the dysregulated expression of integrins and how they regulate related signaling pathways to facilitate the metastatic progression of gastrointestinal cancer, including gastric cancer (GC) and colorectal cancer (CRC), which will address the crucial roles of integrins in gastrointestinal cancer.

## Introduction

In the past decades, cancer incidence and mortality have been rapidly growing worldwide. According to the statistics in 2020 from the American Cancer Society, in both sexes combined, gastric cancer (GC) was the fifth most commonly diagnosed cancer and the fourth leading cause of cancer related death; colorectal cancer (CRC) ranked third in terms of incidence but second in terms of mortality (Sung et al., 2021). Metastasis is the leading cause of gastrointestinal cancer-related death, considered as the most important biological feature of malignant tumors (Guan, 2015). Although increasing efforts have been made to clarify the underlying molecular mechanisms involved in GC and CRC metastatic progression, it is still one of the biggest challenges due to its complexity. Therefore, identifying specific genes governing the metastasis of gastrointestinal cancers will potentially contribute to elucidating mechanisms and discovering early diagnostic biomarkers as well as novel therapeutic targets.

Integrins are a group of transmembrane proteins serve as cell-matrix adhesion receptors for transducing signals and modulating diverse biological processes. To date, 18 α and 8 β subunits have been identified, which can directly form 24 known heterodimers, each α/β combination endows a binding specificity of extracellular domains for different ligands (Hynes, 2002). The subunits, usually around 1,000 (α subunits) and 750 (β subunits) amino acid residues in length, include a membrane-spanning helix, an ectodomain, and a typically short and unstructured cytoplasmic tail, with flexible linker regions between them (Campbell and Humphries, 2011). Integrin activation can regulate their affinity for ligands binding via the conformational changes in the extracellular domain when its C-terminal cytoplasmic tails bind with the activated cytoplasmic proteins, are commonly termed “inside-out” signaling (Springer and Dustin, 2012). Activated integrins determine the downstream signaling events, which highlight the importance of the composition of integrin adhesomes (Horton et al., 2016). Accumulating evidence has demonstrated that this “outside-in” signaling could be also regulated by the sophisticated networks of integrins and other membrane receptors, such as growth factor receptors, urokinase plasminogen activator receptor (uPAR), transforming growth factor-β (TGF-β) receptor, etc. (Giancotti and Ruoslahti, 1999; Zaidel-Bar et al., 2007; Margadant and Sonnenberg, 2010; Kim et al., 2011; Cantor et al., 2015). Altered expression patterns and activities of integrins have been frequently detected in many types of cancers, which could further promote tumor metastasis via downstream signaling pathways (Hamidi and Ivaska, 2018).

The metastatic ability of cancer cells depends on their diverse interactions with surrounding extracellular matrix (ECM) ingredients in the microenvironment (Walker et al., 2018). Integrins are well-known to bind with different ligands such as leukocyte-specific ligands, collagens, laminins, vitronectin, and fibronectin; altered integrins have long been correlated with the metastatic cell behaviors by initiating aberrant cellular signaling (Ganguly et al., 2013). Many researchers have reported modified expression of integrins was frequently observed and participated in metastatic progression of gastrointestinal cancers by multiple mechanisms. Here, we systematically reviewed the integrins that express abnormally in gastrointestinal cancer and the potential mechanisms of certain integrins involved in the multi-steps programmed metastasis including epithelial-mesenchymal transition (EMT), invasion, intravasation, circulation, extravasation and colonization. A deep understanding of integrins-mediated molecular mechanisms and current shortcomings during gastrointestinal cancer metastasis will be necessary for the development of diagnostic and therapeutic strategies against cancer.

## Altered Expression of Integrins in Gastrointestinal Cancer

Aberrant expression of integrins often has been observed in gastrointestinal cancer and received much attention as its fundamental role in cancer malignancy, including uncontrolled proliferation, apoptosis, metastasis, etc. In this section, we first reviewed the altered expression of certain integrins and their involvement in GC and CRC progression (Table 1).

**TABLE 1:** Aberrant expression of integrins in gastrointestinal cancer.

Individual integrins	Altered expression observed in gastrointestinal cancers	Associated phenotypes
α1β1	GC↑, CRC↑	Increased peritoneal dissemination in GC Fukuda et al. (2012). Increased cell proliferation, survival, and migration abilities in CRC Boudjadi et al. (2017).
α2β1	GC↑, CRC↑	Increased cell survival, adhesion, migration, and peritoneal dissemination in GC Lin M.-T. et al. (2007), Chuang et al. (2018). Increased cell anoikis resistance, proliferation, adhesion, metastasis, and stemness in CRC Bartolomé et al. (2014a), Guha et al. (2019), Wu et al., (2019).
α3β1	GC↑, CRC↑	Increased cell adhesion, invasion, and peritoneal dissemination in GC Saito et al. (2010), Chen et al. (2015). Increased cell proliferation, migration, and invasion abilities in HCT-116 CRC cells Tian et al. (2020).
α4β1	GC ↓, CRC↑	Decreased cell invasion and metastatic abilities in GC Park et al. (2004). Increased lymphangiogenesis and lymph node metastasis in CRC Lv et al. (2016).
α5β1	GC↑, CRC↑	Increased angiogenesis, lymph node metastasis, and vascular invasion in GC Ren et al. (2014). Increased cell anoikis resistance and migration; decreased cell autophagy in CRC Guha et al. (2019), Thongchot et al. (2020).
α6β4	GC↑, CRC↑	Increased cell metastasis in GC Gan et al. (2018). Increased cell proliferation, migration, and invasion abilities; decreased cell anoikis in most CRC cells Beaulieu (2019). Increased cell apoptosis in RKO CRC cells Bachelder et al. (1999).
α7β1	GC↑, CRC↑/↓	Increased cell migration, invasion, adhesion, and peritoneal metastasis in GC Yan et al. (2019), Zang et al. (2020). Upregulated α7β1 related with cell invasion and metastasis in CRC Liu et al. (2018). Downregulated α7β1 increased cell proliferation and migration in CRC Li et al. (2018).
α8β1	CRC↓	Increased cell anoikis susceptibility in CRC Benoit et al. (2010).
α9β1	CRC↑	Increased cell proliferation, invasion, and metastatic abilities in CRC Ou et al. (2014).
αvβ3,αvβ5, and αvβ6	GC↑, CRC↑	Increased cell proliferation, migration, and perineural invasion (αv subunit) McCarty (2008), Waisberg et al. (2014), Wang et al. (2019). Increased cell proliferation and invasion abilities (β5 subunit) Shi et al. (2021).

↑, increased; ↓, decreased.

Given the importance of integrins in GC and CRC, the multiple mechanistic roles governing integrins expression were comprehensively discussed. In GC and CRC, the alterations in integrins expression are regulated at different levels, including transcriptional, post-transcriptional, translational, and post-translational level, in which the transcriptional level is most researched. The transcriptional activities of integrin α1, α6, and β4 are positively modulated by the binding of oncogenic Myc to the consensus sequence sites (E box) in the promoter motif of certain integrins in CRC (Ni et al., 2005; Boudjadi and Beaulieu, 2016; Boudjadi et al., 2016; Beaulieu, 2019). Notably, neo integrin α6 is expressed under the form of integrin α6A in CRC cells, where epithelial splicing regulatory protein 2 (ESPR2) is stimulated by Myc alternatively splices α6 (Groulx et al., 2018). The upregulated integrin α1, α6A, and β4 can further activate the RAS/mitogen-activated protein kinase kinase (MEK)/extracellular signal-regulated kinase (ERK) pathway and promote β-catenin signaling to enhance Myc expression, indicating a potential positive feedback loop for sustaining Myc and integrins activity (Boudjadi and Beaulieu, 2016). Furthermore, functional analysis showed that integrins also contain binding sites for Ets, specificity protein (Sp) family transcription factors (TFs) and activating protein-1 (AP-1) in CRC. Li et al. demonstrated that increased expression of integrin β4 in CRC might be regulated by FOSL1 (FOS like 1), an AP-1 transcription factor subunit (Li et al., 2019). The enhanced integrin α3β1 and α5β1 in CRC can be attributed to their increased interaction with activated Ets family TFs, which was induced by the K-ras mediated Raf/MEK/mitogen-activated protein kinase (MAPK) signaling (Schramm et al., 2000). It is worth mentioning that the promoter activity of integrin α5 can be modulated by several other TFs in CRC, such as parathyroid hormone-related protein (PTHrP) (Anderson et al., 2007), zinc finger E-box binding homeobox 2 (ZEB2)-SP1 (Nam et al., 2014), Twist 1 and AP-1 (Nam et al., 2015); while in GC, it has been reported that integrin α5β1 is stimulated by the loss of hypoxia-inducible factor 1a (HIF-1a), an oxygen-dependent transcriptional activator, which results in enhanced metastasis (Rohwer et al., 2008). Besides, Janouskova et al. identified that the reactivation of p53 by Nutlin-3a could specifically inhibit integrin α5β1 expression both at the transcriptional and protein level in colon cancer cells (Janouskova et al., 2013). The TFs involved in regulating individual integrin subunits expression in GC and CRC were summarized in Table 2. As we know, gene transcription is regulated by the interaction between TFs and epigenetic modification (e.g. DNA methylation and histone modifications); the abnormal promoter DNA methylation or histone modifications is another pivotal factor of gene transcription which has been extensively studied. Park et al. reported that the loss of integrin α4 expression was caused by DNA methylation-based transcriptional repression in gastric carcinogenesis (Park et al., 2004). Li et al. identified that hypomethylation of integrin β4 promoter was negatively correlated with down-regulated β4 expression in CRC (Li et al., 2019). Ferraro et al. proved that three methylation at lysine 27 on histone 3 (H3K27me3), was regulated by enhancer of zeste homolog 2 (EZH2), which repressed integrin α2 expression (Ferraro et al., 2013; Ferraro et al., 2014a). However, the effect of other histone modifications, such as demethylation, acetylation or deacetylation, phosphorylation and ubiquitination, on the regulation of integrin expression in gastrointestinal cancer needs further investigation.

**TABLE 2:** Transcription factors involved in the regulation of integrin subunits in GC and CRC.

Individual integrin subunits	Related transcriptional regulation
α1	Myc increases α1 transcriptional activity in CRC Boudjadi et al. (2016).
α2	AP-1 increases α2 transcriptional activity in GC Lin M.-T. et al. (2007).
α3	Ets and Sp family increase α3 transcriptional activity in GC Katabami et al. (2006).
α5	HIF-1a decreases α5 transcriptional activity in GC Rohwer et al. (2008). PTHrP, ZEB2-SP1, Twist1 and AP-1 increase α5 transcriptional activity in CRC Anderson et al. (2007), Nam et al. (2014), Nam et al. (2015).
α6	Myc increase α6 transcriptional activity in CRC Beaulieu (2019).
α7	RAS-responsive element binding protein 1 (RREB1) decreases α7 transcriptional activity in CRC Li et al. (2018). Forkhead box C1 (FOXC1) increases α7 transcriptional activity in CRC Liu et al. (2018).
β1	RelB and nuclear receptor subfamily 4 group A member 1 (NR4A1)/p300/Sp increase β1 transcriptional activity in CRC Hedrick et al. (2017), Zhou et al. (2018). Forkhead box O3 (FOXO3a) increases β1 transcriptional activity in GC Hu et al. (2017).
β3	Homebox D3 (HOXD3) and homeobox B5 (HOXB5) increase β3 transcriptional activity in CRC Yang et al. (2019), Feng et al. (2021).
β4	Myc, zinc-finger with KRAB and SCAN domains 3 (ZKSCAN3), and FOSL1 increase β4 transcriptional activity in CRC Yang et al. (2008), Beaulieu (2019), Li et al. (2019).
β6	Ets proto-oncogene 1 (Ets 1) increase β6 transcriptional activity in CRC Bates et al. (2005).

Moreover, the integrins expression is also post-transcriptionally controlled by microRNA (miRNA) in GC and CRC. As examples, miR-21 inhibits integrin β4 expression in CRC (Ferraro et al., 2014b) and miR-30a suppresses integrin α2 expression in intestinal-type early gastric carcinogenesis (Min et al., 2020). However, the role of other regulatory factors (e.g. RNA-binding proteins, lncRNAs and circRNAs) in integrin mRNAs regulation at the post-transcriptional level has not been reported in GC and CRC. Of note, recent studies have revealed that the stability, splicing and nuclear export of mRNA were regulated by RNA modifications, especially the N6-methyladenosine (m^6^A) modification, which eventually regulates the mRNA translation (Jin et al., 2019; Li E. et al., 2020). For example, integrin α6 has been proved to be regulated by m6A post-transcriptionally in bladder cancer development (Jin et al., 2019). Prospectively, further explorations of RNA modifications on integrins in gastrointestinal cancer are expected. For the translation level, Cantor et al. showed that the upregulated integrin β6, which was modulated by eukaryotic translation initiation factor 4E (eIF4E), may initiate a cascade of downstream signaling promoting CRC metastasis (Cantor et al., 2015). Kline et al. illustrated that Src activation decreased integrin α3 expression at the protein level, but not the mRNA level, in a MAPK-dependent manner in CRC (Kline et al., 2009). Finally, as we know that post-translational modifications (PTMs) are also critical mechanisms to increase proteomic diversity. Although increasing evidence indicated that integrins are regulated by several types of PTMs, including phosphorylation, glycosylation, ubiquitination, nitrosylation and acetylation (Goldfinger et al., 2003; Oxley et al., 2008; Lobert et al., 2010; Meves et al., 2011; Isaac et al., 2012; Hou et al., 2016; Hang et al., 2017; Marsico et al., 2018; Gahmberg et al., 2019; Vega et al., 2020), how these PTMs regulate the stability and interactome of individual integrins are poorly understood, especially in gastrointestinal cancer. Therefore, more studies are needed to understand the dynamics of these PTMs, how they coordinately regulate integrins, the functional consequences of these PTMs and which is cancer metastasis related. 

## Roles of Integrins in Gastric Cancer Metastasis

### Integrins and Epithelial-Mesenchymal Transition

**FIGURE 1:**
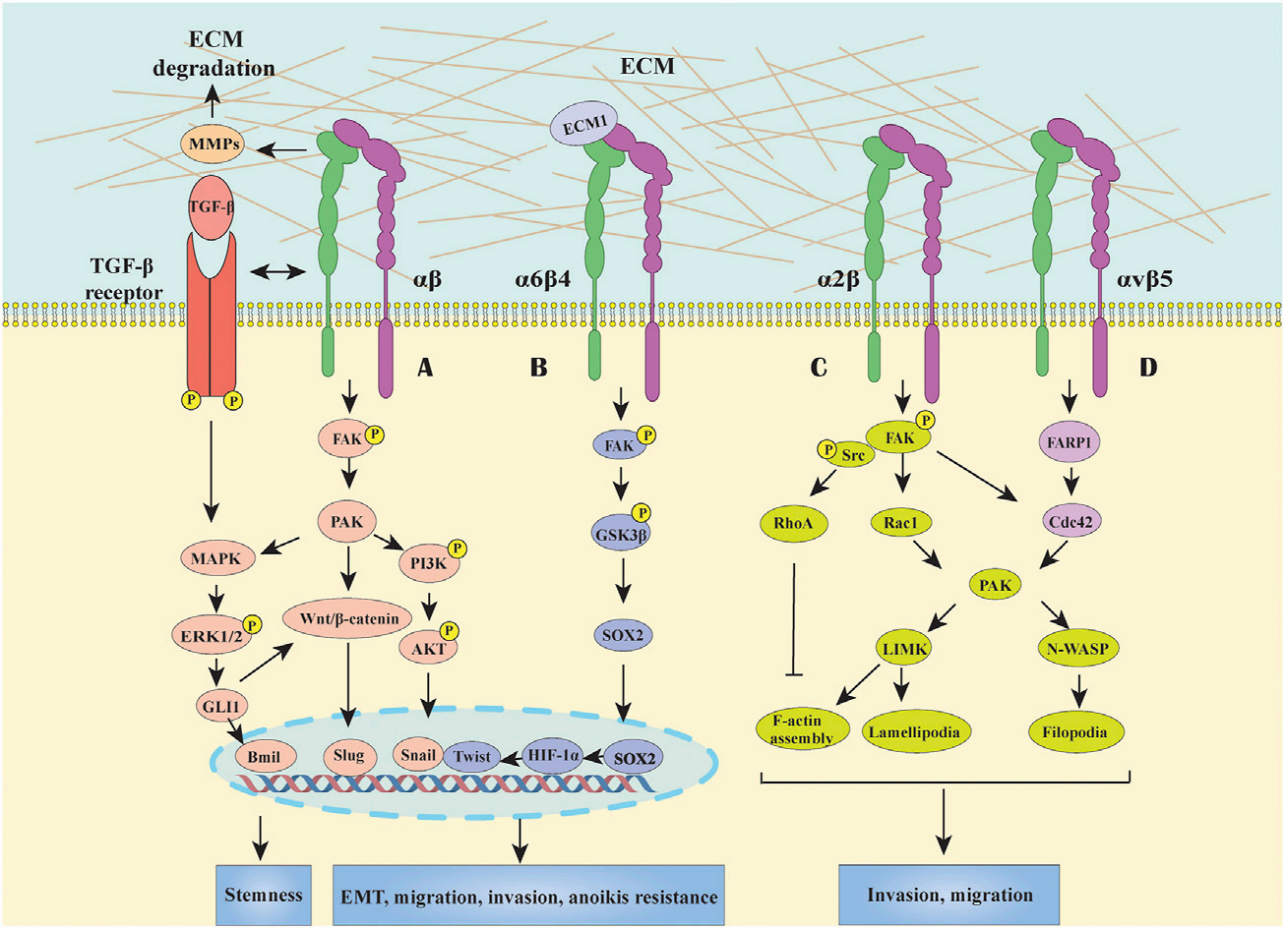
Integrins mediated signal transduction pathways in GC. **(A)** Most integrins initiate FAK-generated signaling, including MAPK/ERK, PI3K/AKT and Wnt/β-catenin pathways to participate in EMT, migration and invasion processes and acquire stemness and anoikis resistance. Besides, the crosstalk between TGF-β and integrin signaling also can activate downstream effectors which cooperatively modulate EMT in a cross-regulation manner in GC. **(B)** Integrin α6β4 activate the FAK/GSK3β signaling pathway, inducing the expression of transcription factor of SOX2, HIF-1α, Snail and Twist, to promote anoikis resistance and EMT activation. **(C)** Integrin α2 can stabilize F-actin, promote filopodia formation and lamellipodial protrusion by upregulating RhoA, Rac1, and Cdc42, thus facilitating GC cell migration. **(D)** Association of integrin αvβ5 and FARP1 can facilitate cell motility by boosting the downstream molecule Cdc42 activity.

EMT has been considered as a critical component of the metastatic program changing cell morphology and enhancing cancer cell mobility and invasion abilities (Aiello and Kang, 2019). Substantial evidence showed that altered expression of integrins correlated with GC metastasis, and dysregulated integrin-mediated signaling pathways, such as focal adhesion kinase (FAK)/p21-activated kinase (PAK), Wnt/β-catenin and FAK/glycogen synthase kinase-3β (GSK3β), played essential roles in EMT process. Here, we systemically summarized the aberrant signaling events triggered by integrins during EMT in GC (Figure 1).

It is widely acknowledged that FAK is a major signal transduction downstream molecule of integrins which could affect EMT processes, including downregulation of cell-cell adhesion and overcome anoikis. As we know, FAK is comprised of three main domains: the N-terminal FERM domain, the central kinase domain and the C-terminal focal adhesion targeting (FAT) domain (carrying six phosphorylated tyrosine sites) (Kokkinos et al., 2007; Murphy et al., 2020). In normal circumstances, the interaction between the FERM and kinase domain maintains an autoinhibited state, and the autophosphorylation site Y397 is among the linker between them (Tapial Martínez et al., 2020). Once the cytoplasmic tail of the integrin β subunit binds to the amino terminus of FAK, Y397 autophosphorylation converts the site into a high-affinity binding site for the SH2 domain of Src resulted in Src activation, which in turn phosphorylates other tyrosines of FAK, thus inducing the complete catalytic activity of FAK, consequently recruiting other signaling molecules to the focal adhesion sites and sustaining the signaling (Cooper et al., 2003; Tapial Martínez et al., 2020). The mechanisms of FAK in triggering EMT in GC are proposed to serve as the intersection of multiple signal pathways, including MAPK, phospoinositide 3-kinase (PI3K)/AKT and Wnt/β-catenin (Matsuoka et al., 2012; Dammann et al., 2014; Wang et al., 2019). Indeed, these integrins/FAK-mediated signalings in GC also contribute to overcoming apoptosis, which is a typical characteristic of the acquisition of mesenchymal phenotype. For instance, it has been shown that the interaction between integrin β4 and extracellular matrix protein 1 (ECM1) could activate the β4/FAK/GSK3β signaling pathway, then induced the expression of transcription factor SOX2 and HIF-1α, which eventually contribute to EMT (Gan et al., 2018) (Figure 1). Besides, enhanced PI3K/AKT, MAPK and Wnt/β-catenin signals can stimulate anti-apoptotic proteins such as Snail, Twist, and etc., thus promoting anoikis resistance and EMT activation (Paoli et al., 2013; Peng et al., 2014) (Figure 1). Moreover, Integrin β1 could associate with the carbohydrate-recognizing domain (CRD) of Galectin-1 (Gal-1) secreted by activated cancer-associated fibroblasts (CAFs) via its extracellular carbohydrate structure, resulting in Gli1 expression which may further activating Wnt/β-catenin signaling, and finally trigger the EMT process in GC cells (Elola et al., 2005; Chong et al., 2016; Zhang et al., 2020). In fact, not only Gal-1 upregulation, but also the alterations in glycosylation pattern of its binding protein (e.g. integrins) contribute to EMT process in GC (Kariya et al., 2017). For instance, N-acetylglucosaminyltransferase III (GnTIII) and GnTV overexpression-modified glycosylation of integrin α3β1 and E-cadherin could induce EMT and cell invasion in GC cells, highlighting that targeting specific glycosylation might have potential in anti-cancer therapy; however, this needs further investigation (Zhao et al., 2006; Pinho et al., 2013).

In addition, TGF-β as the most studied growth factor in EMT can regulate the expression and activation states of certain integrins and exert a synergistic effect with integrin signalings. For example, TGF-β-induced signaling activated integrin β1 by phosphorylating its cytoplasmic tail in hepatocellular carcinoma invasion (Fransvea et al., 2009), while TGF-β1 can increase the expression of integrin α2/α3 and then facilitate GC cell spreading and migration (Lee et al., 2005), suggesting a tissue- and cell-specific regulatory manner which need to be further investigated. Besides, αv-containing integrins can drive latent TGF-β activation to sustain EMT by interacting with arginine–glycine–aspartic acid (RGD) motif on TGF-β propeptide, a member of the inactive TGF-β complexes (Ludbrook et al., 2003; Annes et al., 2004). In contrast, the effect of integrin αv on EMT in GC have not been investigated. Moreover, the crosstalks between TGF-β and integrin signalings can activate downstream effectors resulting in EMT, tumor invasion and metastasis (Mamuya and Duncan, 2012). For instance, the activated-integrin/FAK signal further mediate the activation of downstream molecules (e.g. MAPK, PI3K/AKT, and Ras); meanwhile, TGF-β signaling can activate SMAD, MAPK, and PI3K signalings, which therefore modulate EMT in a cooperative manner in GC.

### Integrins in Migration and Invasion

Cell migration and invasion are highly complex processes, in which integrins-mediated signalings control the organization of actin cytoskeleton via FAK/Src-activated small Rho GTPases, including RhoA, Rac, and Cdc42 (Huttenlocher and Horwitz, 2011) (Figure 1). As an example, integrin α2 can stabilize F-actin, promote filopodia formation and lamellipodial protrusion by upregulating Rac1 and Cdc42, thus facilitating GC cell migration (Chuang et al., 2018). Recent research reported that the association between integrin αvβ5 and pleckstrin domain protein 1 (FARP1) facilitated cell motility and filopodium formation of GC cells by activating the downstream molecule Cdc42 (Hirano et al., 2020). In detail, the crystal structure of FARP1 exhibited an autoinhibited conformation in which the RhoGTPase-binding site of the DH and the first PH (PH1) domain is primarily blocked by the second PH domain (PH2) (Kuo et al., 2018). Importantly, this autoinhibition is canceled once integrin αvβ5 binds to FARP1, further promoting cell migration and invasion through Cdc42/PAK signaling pathway (Chuang et al., 2018; Hirano et al., 2020). Considering the complex spatial structure of both integrin αvβ5 and FARP1, the mechanism of how they regulate each other’s activity needs further investigation.

Peritoneal metastasis (PM) appears to be one of the most frequent route of metastasis or recurrence in patients with GC and is usually associated with poor prognosis (Rau et al., 2020). Previous works defined integrins as crucial cell adhesion molecules involved in the adhesion of exfoliated GC cells to the mesothelium, which serve as an essential step in the initial PM process. It was reported that the interaction between integrin α3β1 and laminin-5 potentiated the cell adhesion to the peritoneum and the production of matrix metalloproteinase-9 (MMP-9), which facilitated PM by the degradation of ECM in GC (Saito et al., 2010). Specifically, laminin-5, which was produced by mesothelial cells, could be recognized or modulated by integrin α3β1 via the following domains: the LG domains of α chain, C-terminal short stretch of β chain and nearly C-terminal glutamic acid residue of γ chain (Yamada and Sekiguchi, 2015). Moreover, integrin α2β1-mediated the cysteine-rich angiogenic inducer 61 (CYR61)/AP-1 cascade, could promote cell adhesion to the peritoneum (Lin M.-T. et al., 2007). In addition, the activated ERK/JNK signaling and upregulated integrin α5 and fibronectin expression, which is induced by the association of vascular endothelial growth factor A (VEGFA) with VEGF receptor 1 (VEGFR1) under hypoxic microenvironment, could promote PM in GC (Wang et al., 2020). Furthermore, integrin αvβ3 was reported to promote the PM in GC cells, of which the potential αvβ3/ERK/GLI1 pathway-mediated maintenance role in stem cell-like phenotype of exfoliated cells was involved (Dong et al., 2019). A recent study showed the lipid phosphate phosphatase-related protein type 4 (LPPR4) could upregulate integrin α subunits (including α1, α2, α5, α6, and α7), but not β subunits, expression via SP1 transcription factor; thus, activating the FAK/Src/AKT/MMP2 signaling pathway, which eventually promoted the PM in GC cells (Zang et al., 2020). Overall, these findings emphasized the importance of integrins in PM, suggesting certain integrins may serve as a promising diagnostic marker and treatment strategy for GC patients with PM, and more clinical correlations are needed to validate these possibilities.

### Integrins in Intravasation, Circulation, Extravasation and Colonization

Increasing evidence implicated integrins played key roles in the remaining metastatic steps since tumor cells enter the blood vessels, including intravasation, circulation and extravasation, leading to colonization at a distant site (Hamidi and Ivaska, 2018). However, there is limited research about the role of integrins in these steps in GC. It has been demonstrated that CYR61 can induced C-X-C chemokine receptors CXCR1/CXCR2 expression by activating integrin αvβ3/Src/PI3K/AKT pathway, thus exhibiting increased potency in interleukin-8 (IL-8) chemotaxis, transendothelial migration and intravasation in GC cells (Lin B.-R. et al., 2007). In addition, integrin β4 and its distinctive effects on regulation of cytoskeletal and hemidesmosomes have been well studied. Under physiological conditions, integrin β4 forms the complex with plectin (PLEC), which can maintain filamin A (FLNA) and cytoskeleton stability, once the integrin β4-PLEC interaction is disrupted, the disassociated PLEC binds to F-actin and damages the cytoskeleton network (Koster et al., 2003). In GC cells lacking transmembrane protein 268 (TMEM268), a novel protein involved in tumorigenesis, lead to increased ubiquitin-mediated degradation of integrin β4 and cytoskeleton remodeling, thus losing the possibility of circulating tumor cells (CTCs) adhere to vascular endothelium, extravasate into other organs, and eventually fail to form metastatic colonization (Hong et al., 2019). Accordingly, a recent study found that the immunostaining intensity of integrin β4 in lymphovascular invasion (LVI) and perineural invasion (PNI) in GC was significantly higher than that in normal stomach, indicating β4 may be a potential novel marker for detection and diagnose of LVI and PNI in GC patients (Li J. et al., 2020).

During the metastatic process, extravasation is a critical step for CTCs to form the pre-metastatic niche and its efficiency is largely dependent on the permeability and integrity of the vascular endothelium (Lambert et al., 2017). In many types of cancers, increased angiopoietin 2 (Ang2) accompanied by the decreased angiopoietin receptor Tie2 exhibit the predisposition to the integrin α5β1-Ang2 interaction, which has been implicated in compromised permeability through integrin β1 signaling (Imanishi et al., 2007; Hakanpaa et al., 2015). Of note, the endothelial integrin α5 could also *trans*-interacted with neuropilin 2 (NRP2) on cancer cells, promoting the vascular extravasation in pancreatic adenocarcinoma mouse xenograft models (Cao et al., 2013). Although upregulated expression of integrin α5β1 correlates with increased metastasis and vascular invasion in GC as mentioned above, the mechanisms involved in its role in extravasation and metastasis are poorly understood and need further investigation.

## Functions of Integrins in Colorectal Cancer Metastasis

### Integrins and Epithelial-Mesenchymal Transition

**FIGURE 2:**
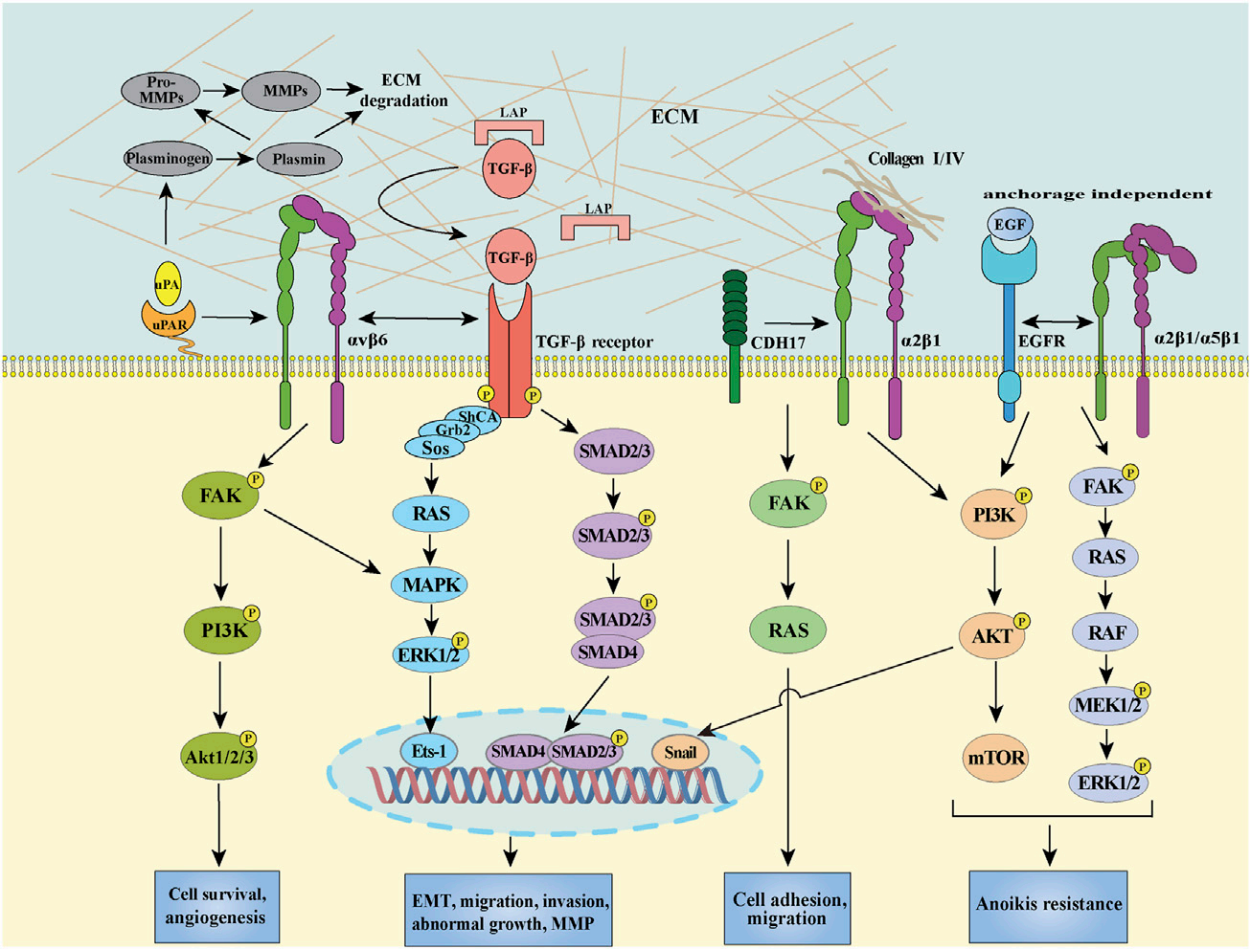
Schema of integrin αvβ6/TGF-β receptor/uPAR, integrin α2β1/CDH17, integrin α2β1 (or α5β1)/EGFR mediated signal transduction pathways in CRC. Integrin αvβ6 separately interacts with uPAR and TGF-β receptor, initiating intracellular signaling contributes to the activation of transcription factors, such as Ets-1 and SMAD2/3/4 through the FAK/PI3K, RAS/ERK, and SMAD pathways. CDH17 provokes a conformational change in the β1 subunit, activating the downstream FAK/RAS pathway to increase cell adhesion. The crosstalk between integrin α2β1/α5β1 and EGFR can induce PI3K/AKT signaling to promote EMT through stimulating transcription factor snail in CRC cells. On the other hand, integrin α2β1/EGFR also can activate FAK/ERK and PI3K/AKT survival pathway resulting in anoikis resistance in the absence of ECM.

Abundant evidence show that EMT is associated with the invasive or metastatic phenotype in CRC (Vu and Datta, 2017). Integrins have long been known to regulate cell-cell and cell-ECM events and trigger the downstream signaling pathways, leading to malignancy. Among the multiple integrins, integrin αvβ6 has been well studied about its role in EMT initiation and progression in CRC. Activated TGF-β signaling plays essential role in the modulation of integrin αvβ6 level via promoting SMAD4 binding to its promoter motif, thus facilitating cell migration (Bandyopadhyay and Raghavan, 2009). Of note, integrin expression can also be regulated by the non-canonical pathway (SMAD independent manner) induced by TGF-β, for example, ERK/MAPK signal pathway is involved in the TGF-β-triggered integrin αvβ6 transcription via Ets-1 (Callaway et al., 2006). In addition to TGF-β, interleukin-6 (IL-6) was reported to induce EMT by increasing integrin β6 expression in CRC cells, during which the IL-6 receptor/STAT3 signaling pathway was involved (Sun and Shang, 2020). Interestingly, integrin αvβ6 can mediate latent TGF-β activation by directly interacting with the RGD sequence presented in the latency-associated protein (LAP), which require the latent TGF-β binding protein-1 (LTBP-1) to localize, concentrate and fix the latent form of TGF-β (Annes et al., 2004). Moreover, the integrin αvβ6-mediated TGF-β activation can further stimulate fibroblasts to secrete stromal cell-derived factor-1 (SDF-1), resulting in CRC invasion via the SDF-1/C-X-C chemokine receptor type 4 (CXCR4) axis (Peng et al., 2018). Therefore, upregulated integrin αvβ6 could cooperate with TGF-β to induce and sustain EMT process, providing a positive feedback loop to perpetuate EMT and rendering the tumor microenvironment more amenable to form the pre-metastasis niche (Figure 2). Given the importance of integrin αvβ6 in EMT and metastasis, it is reasonable to speculate that αvβ6 may be a potential marker of EMT as well as a novel therapeutic target for CRC in the near future.

In addition to integrin αvβ6, other integrins can also contribute to EMT to accelerate migration in CRC. Wu et al. demonstrated that the interaction between integrin α2β1 and enriched collagen I could activate the PI3K/AKT signaling pathway to induce the EMT process through transcription factor snail activation in CRC cells (Wu et al., 2019) (Figure 2). Significantly, blocking integrin α2β1 efficiently suppressed the metastasis and combination of α2β1 inhibitor with chemotherapeutic agents exhibited a synergistic antitumor effect, revealing a potentially promising treatment approach for CRC treatment (Wu et al., 2019). Recently, increasing evidence highlighted that the expression of integrins in tumor budding (TB), a process that exhibits characteristics of partial EMT, may predict survival in patients with CRC. For instance, Zhou et al. observed that the interaction between integrin β1 and laminin-5γ2 promoted the TB via FAK and Yes-associated proteins (YAP) activation in CRC (Zhou et al., 2020). Slik et al. demonstrated that aberrant EMT-associated markers, including integrin β4, E-cadherin and zonula occludens-1 (ZO-1), could be detected by multiplex immunohistochemistry in tumor buds of stage II CRC, highlighting the potential role of integrins in EMT-like phenotypes of TB (Slik et al., 2019). Moreover, transmembrane protease serine 4 (TMPRSS4) was reported to suppress E-cadherin expression, leading to EMT and invasiveness via stimulating integrin α5β1 expression in CRC (Kim et al., 2010). Of note, several other integrins, such as integrin αvβ3, α6β4, and β5, have been demonstrated to play crucial roles in EMT of different types of cancer, including breast cancer, hepatocellular carcinoma and renal cell carcinoma (Bianchi et al., 2010; Knowles et al., 2013; Mori et al., 2015; Li et al., 2017; Kariya et al., 2021), the effect of these integrins on EMT in CRC need to be explored. In addition, how about the impact of integrins on other EMT inducers, such as EGF, Wnt and Hedgehog (Hh), -mediated EMT, and especially how integrins crosstalk with the membrane receptors of these inducers as well as TGF-β receptors are still unaddressed.

### Integrins in Migration and Invasion

Integrin αvβ6 is thought to regulate several metastatic phenotypes in CRC via diverse mechanisms (Cantor et al., 2015). uPAR is a GPI anchored protein, which lacks transmembrane and intracellular domains and requires cooperation with other transmembrane receptors to mediate signal transduction (Smith and Marshall, 2010). The crosstalk between integrin αvβ6 and uPAR is implicated in the regulation of their downstream signalings upon urokinase (uPA) binding (Figure 2) (Smith and Marshall, 2010; Sowmya et al., 2014). Mechanistically, integrin αv could interact with the outer surface of uPAR domain III region, which provides structural support and/or shield the DI-DII linker region of uPAR from the cleavage by pericellular proteolysis, thus promoting the persistence of the active binding conformation of uPA on cell surface (Cantor et al., 2015). The increased association between integrin αvβ6 and uPA/uPAR could further induce MMP-9 secretion, thus prompting the degradation of extensive ECM components in a MAPK/extracellular signal-regulated kinase (MEK1) dependent manner (Gao et al., 2014; Cantor et al., 2015). In parallel to the uPAR signaling, TGF-β could also be activated by integrin αvβ6 as mentioned above, highlighting the importance of integrin αvβ6 during multiple metastasis steps. Furthermore, upregulated integrin αvβ6 was involved in IL-8-promoted migration in CRC (Sun et al., 2014). Given that integrin αvβ6 provides a structural foundation to facilitate the recruitment of TGF-β, IL-8 and critical components of the PA cascade, a pericellular interactome has been proved to play critical roles in concentrating key metastasis-related proteins and activating downstream signaling axis (Figure 2). In addition to integrin αvβ6, integrin β1-uPAR complex could also enhance the CRC cell migration and invasion, as well as ECM degradation by activating ERK/MAPK pathway and increasing MMP-2/MMP-9 expression (Ahmed et al., 2003).

The extensive molecular crosstalk between integrins and receptor tyrosine kinases (RTK) makes the metastasis mechanism more complicated. Guha et al. found that integrin α2β1/α5β1 could colocalize with EGFR on cell surface in the anoikis-resistant CRC cells which was involved in the migration of CRC cells from the primary site to newly distant site(s) and could grant the cells with stem cell-like properties; the integrin α2β1/α5β1-EGFR complex can activate the ERK/AKT-mediated survival pathway and inhibit caspase-3 activation and consequently inducing anoikis resistance in the absence of ECM (Guha et al., 2019). Additionally, integrin α6β4 could also modulate the metastatic process of CRC via cooperating with RTKs and activating oncogenic signaling. This crosstalk can phosphorylate the β4 cytoplasmic tail via stimulating the Src family kinases (SFKs), triggering the ERK/PI3K pathway to regulate specific TFs and eventually facilitating the cell migration. As mentioned above, integrin α6 subunit can regulate the downstream effector Myc, which appears to promote the transcription of integrin α6β4 via the Wnt/β-catenin pathway (Beaulieu, 2019). Of note, the role of certain integrin in CRC cell migration remains controversial. For instance, it has been shown that the morphological response of CRC cells on laminin-10, which contributes to cell adhesion and spreading, is mediated by the synergistic action of EGFR in an integrin α3β1 dependent manner (Pouliot et al., 2000). However, Hashida et al. demonstrated that integrin α3β1 could form complexes with MRP-1/CD9 and KAI1/CD82, which was negatively correlated with CRC progresses (Hashida et al., 2002). Thus, the exact role of integrin α3β1 in CRC cells has not been fully understood and further investigations are needed to address whether the function of integrin α3β1 in CRC is cell line dependent through specific binding partners.

Moreover, integrins could promote CRC migration and invasion by interacting with other proteins, such as cadherin-17 (CDH17), CYR61, glucose-regulated protein 78 (GRP78), and periostin (PN). In detail, Bartolomé et al. demonstrated that the integrin α2β1 expression was highly associated with liver metastasis in CRC, especially in the patients with late-stage metastasis, mechanistically, CDH17 was involved in binding with and activate integrin α2β1 through its RGD motif and leading a conformation change of integrin β1, which activate the MAPK signaling pathway to induce CRC cell adhesion, in the meanwhile, CDH17-activated integrin α2β1 can interact with collagen IV in a RGD independent manner, which further enhances the cell adhesion to collagen IV and thus increasing cell proliferation ability (Bartolomé et al., 2014a; Bartolomé et al., 2014b) (Figure 2). Monnier, et al. showed that integrin αvβ5 can cooperate with matricellular protein CYR61 to enhance CRC cell invasion and metastasis in the presence of preirradiated stroma (Monnier et al., 2008). Furthermore, Li et al. illustrated that the interplay between integrin β1 and GRP78 could directly facilitate CRC cell migration and invasion (Li et al., 2013). Recently, Thongchot, et al. showed that PN could control the autophagy-regulated cell migration through binding to integrin α5β1 or α6β4 and sequentially activating the AKT pathway (Thongchot et al., 2020). In addition to these outer membrane regulations, the inner membrane regulation was also involved. For example, Beaulieu identified that enhanced integrin α6β4 can preferentially interact with cytoskeletal keratins of hemidesmosomes in the cytoplasm, resulting in the acquisition of a more migratory and anoikis-resistant phenotype in CRC cell (Beaulieu, 2019). Although the importance of these integrin-associated complexes has been highlighted in CRC cell migration and invasion, the detailed mechanisms of how they interplay with each other, especially in different pathological conditions, are still unclear, which need follow-up investigations.

### Integrins in Extravasation and Colonization

Extravasation is a process of CTCs arresting, adhering, and passing through vascular endothelium after circulation (Azevedo et al., 2015). It has been demonstrated that the P-selectin binding-mediated activation of integrin α5β1 can promote cell adhesion to endothelium via the PI3K and p38 MAPK signaling pathways in CRC cells (Reyes-Reyes et al., 2006). In addition to integrin α5β1, the integrin αvβ5 could be activated by autocrine TGF β-induced in CRC cells, enhancing integrin αvβ5/Src signaling and then inducing the dissociation of VE-cadherin junctions between endothelium cells which facilitated extravasation (Ma et al., 2008). Furthermore, integrin αvβ5 could also bind to fibronectin and promote the adhesion of CRC cells to endothelium; however, it was not involved in the following metastasis step of transendothelial migration into the liver parenchyma (Enns et al., 2005). Moreover, the adhesive and invasive of colorectal CTCs within the hepatic microvasculature by intravital fluorescence microscopy showed that integrin α2, α6, β1, and β4 mediate the extravasation of CRC cells into liver (Enns et al., 2004). Functional blocking of integrin α2, α6, and β4 in HT29 CRC cells could inhibit the extravasation process (Robertson et al., 2009), further highlighting the importance of different integrins during the extravasation of CRC cell. However, the detailed molecular mechanism on how these integrins-mediated extravasation, especially the precise balance between the dissociation and adhesion, remains unclear. For the colonization process, the integrin α6/E-cadherin supramolecular complex was reported to strengthen their binding ability to hepatic angiopoietin-like 6, driving the liver homing and colonization of CRC cells (Marchiò et al., 2012). However, the role of integrin in intravasation and circulation during metastasis in CRC is still unclear and needs more investigation. Given that high expression of these integrins are usually associated with poor survival in metastatic CRC, more studies should focus on the specific clinical significance of the indicate integrins as biomarkers and therefore develop potential inhibitors in personalized treatment to improve CRC patient survival.

## Clinical Trials of Integrin-Related Targets in Gastrointestinal Cancer

Several hundred drugs targeting integrins have been identified. The integrin-targeted drugs licensed by some companies may vary from target indications and development stages, for example, some drugs are discontinued in one indication but are tested in others (Raab-Westphal et al., 2017). Here, we conclude the latest stage of clinical trials of integrin-related drugs in gastrointestinal cancer treatment. For example, the combination of the abituzumab (EMD 525797), a monoclonal inhibitory antibody targeting αv integrins, with irinotecan and cetuximab were tested in K-ras wild-type metastatic colorectal cancer patients, however, failed their primary endpoints and ended in phase 2 trial (withdrawal study, ClinicalTrials.gov Identifier: NCT03688230). In addition, the humanized monoclonal antibodies etaracizumab and MEDI522, which directed against the human αvβ3 integrin, were tested in the patients with irinotecan-refractory advanced colorectal cancer and have completed its phase 2 trial (ClinicalTrials.gov Identifier: NCT00284817 and NCT00027729).

Although the clinical treatment result is less encouraging, the potential diagnostic value of targeting integrin seems promising. For instance, in monitoring efficacy and adverse events of apatinib in malignancies (e.g. stomach cancer), the 18F-ALF-NOTA-PRGD2, which can highly combine with integrin αvβ3, has completed its phase 4 trial in the monitoring of the antiangiogenic status, and finally propose to evaluate the feasibility of 18F-RGD PET/CT (ClinicalTrials.gov Identifier: NCT03384511). Furthermore, the 18F-αvβ6-binding-peptide, a radiotracer, for imaging patients with primary tumor or other sites of metastasis (e.g. lung, breast, and colorectal, or pancreatic) is under the early phase 1 trial (ClinicalTrials.gov Identifier: NCT03164486), which may significantly improve the ability to locate the tumor in the patient.

Considering RGD sequence serves as the primary recognition domain in multiple integrins-ECM interactions, such as integrin α5β1, αvβ3, αvβ5, and αvβ6, many researchers have been focused on designing and optimizing the synthetic RGD binding ligands to target certain integrins (Gurrath et al., 1992; Janssen et al., 2002; Meyer et al., 2006; David et al., 2018). Of note, combination of the small molecule antagonist cilengitide, an RGD-mimetic cyclicized pentapeptide which target integrin αvβ3 and αvβ5, with temozolomide and radiation therapy has completed the phase 3 clinical trials in patients with newly diagnosed glioblastoma (ClinicalTrials.gov Identifier: NCT00689221). Nonetheless, the combination of cilengitide with chemotherapy exhibited promising prospects in advanced non-small-cell lung cancer (Vansteenkiste et al., 2015); therefore, application discovery for synthetic integrin ligands in gastrointestinal cancer treatment remains to be investigated.

## Conclusion and Perspective

Taken together, aberrant expression of integrins contribute to several metastatic steps including EMT/invasion, intravasation, circulation, extravasation, and colonization in gastrointestinal cancer. In a mechanistic manner, interaction/crosstalk between different ligands (e.g. laminin-5, collagen IV, and fibronectin) or transmembrane receptors (e.g. uPAR, TGF-β, and EGFR) and integrins mediate the conformational rearrangement-dependent activation, which induces a series of downstream pathways to promote gastrointestinal cancer metastasis. Recent findings provide a theoretical basis for the potentiality of integrins as novel diagnostic markers and therapeutic targets for gastrointestinal cancer. Compared to their promising achievements in diagnosis, the therapeutic value of targeting integrins needs further investigation. Specifically, nascent inhibitory peptides, anti-integrins monoclonal antibodies and the combination with other therapeutic approaches (e.g. antibody-drug conjugates, nanoparticle-based delivery, and RNA interference technology) are encouraged to be developed and investigated in clinical trials.

Gastrointestinal cancer exhibits higher propensities to metastasize to the liver, lymph nodes, peritoneal, and then subsequently spread to the lung or other organs (Chen et al., 2012), indicating that a limited number of organs provide a suitable stromal environment for their colonization. It is worth mentioning that there has been an increasing interest in exploring the function of exosomal integrins (including integrin α6β4, α6β1, α2β1, αMβ2, αvβ3, αvβ6, and α5β1) on metastasis, especially the organotropic metastasis, in breast cancer, pancreatic cancer, prostate cancer, and lung cancer metastatic models (Bijnsdorp et al., 2013; Fedele et al., 2015; Hoshino et al., 2015; Singh et al., 2016; Hurwitz and Meckes, 2019; Li X. et al., 2020; Gaballa et al., 2020; Casari et al., 2021; Chen et al., 2021; Wu et al., 2021). However, the essential exosomal integrins involved in gastrointestinal cancer metastasis have not been identified yet. In addition, further studies are needed to focus on the detailed mechanisms involved in exosomal inegrins-mediated pre-metastatic niche evolution and investigate the potential of exosomal integrin(s) as a marker and driver of cancer metastasis, especially in gastrointestinal cancer metastasis. Clinically, a study titled “Identification of New Diagnostic Protein Markers for Colorectal Cancer (ClinicalTrials.gov Identifier: NCT04394572)”, which include focusing on the specific integrins derived from circulating tumor exosomes in the context of colorectal cancer to evaluate the diagnostic performances of related markers, is under the recruiting stage.

Notably, metastatic latency is a clinical phenomenon for many types of cancer, including gastrointestinal cancer, mainly due to cancer cell dormancy. Metastatic dormancy is defined by a relatively long disease-free interval (months, years or even decades, differ from cancer to cancer) between successful therapy or removal of the primary tumor and subsequent clinical relapse with disseminated disease (Anderson et al., 2019). Dormant tumor cells usually exhibit resistance to chemotherapy due to their arrested cell cycle (Naumov et al., 2003) or the protection by the cellular molecules and extracellular components of their microenvironment (Sethi et al., 1999; Weaver et al., 2002; Ghajar et al., 2013). Recently, the factors that maintain tumor cell dormancy in the pre-metastatic niches have been unraveled, including different ECM components, cytokines and other proteins (Anderson et al., 2019), however, about how dormancy is broken remains less understood. Intriguingly, integrins β1, α3β1, and α4β1 have been implicated in the reactivation of the dormant breast cancer cells (Shibue and Weinberg, 2009; Chen et al., 2011; Shibue et al., 2012; Shibue et al., 2013; Albrengues et al., 2018), highlighting the importance of integrins-mediated adhesion signaling between metastasis-initiating cells and perivascular niches in metastatic dormancy and reactivation. Therefore, more studies are needed to focus on the role of indicated integrin in gastrointestinal cancer metastatic dormancy and reactivation.

In summary, integrins-mediated gastrointestinal cancer metastasis is a complex and multi-step process. The very concept of designing a metastasis-specific therapeutic should consider which step of the process is best to target, and targeting any stage of the metastatic process requires a cancer-specific understanding of the mechanisms involved. We therefore prospectively suggest the following points need to be addressed in further investigations: 1) the dynamics of PTMs, especially glycosylation on integrins during gastrointestinal cancer metastasis; 2) the integrin-associated mechanisms linking immune system/metabolism and metastasis, which is better to understand the integrin dependency; 3) patient-derived xenograft and related genetically engineered mouse models will be helpful to explore truly effective agents that block the integrin-specific signaling; and 4) integrins-related RNAi and nanoparticle formulations need to be studied during gastrointestinal cancer metastasis.
